# Improved Relapse-Free Survival in Patients With High Natural Killer Cell Doses in Grafts and During Early Immune Reconstitution After Allogeneic Stem Cell Transplantation

**DOI:** 10.3389/fimmu.2020.01068

**Published:** 2020-05-29

**Authors:** Lia Minculescu, Anne Fischer-Nielsen, Eva Haastrup, Lars Peter Ryder, Niels Smedegaard Andersen, Ida Schjoedt, Lone Smidstrup Friis, Brian Thomas Kornblit, Søren Lykke Petersen, Henrik Sengelov, Hanne Vibeke Marquart

**Affiliations:** ^1^Department of Clinical Immunology, Copenhagen University Hospital, Rigshospitalet, Copenhagen, Denmark; ^2^Department of Hematology, Copenhagen University Hospital, Rigshospitalet, Copenhagen, Denmark; ^3^Department of Clinical Medicine, Copenhagen University, Copenhagen, Denmark

**Keywords:** natural killer cells, innate effector cells, allogeneic transplantation, stem cell graft, immune reconstitution

## Abstract

Mature immunocompetent cells from the stem cell graft as well as early robust immune reconstitution are essential for the graft-vs. -tumor (GVT) effect to eliminate residual malignant cells after allogeneic hematopoietic stem cell transplantation (HSCT). In this prospective study we characterized graft composition of T- and NK cell subsets in 88 recipients of peripheral blood stem cell grafts with multicolor flowcytometry. Our primary aim was to analyze the impact of graft composition on immune reconstitution and clinical outcomes after transplantation. Patients transplanted with graft NK cell doses above the median value of 27 × 10^6^/kg had significantly increased relapse-free-survival compared to patients transplanted with lower doses, HR 2.12 (95% CI 1.01–4.45, *p* = 0.04) Peripheral blood concentrations of NK cells obtained from donors before G-CSF mobilization were significantly correlated to graft NK cell doses (Spearman's ρ 0.53, *p* = 0.03). The dose of transplanted NK cells/kg correlated significantly with NK cell concentrations in patients early after transplantation (Spearman's ρ 0.26, *p* = 0.02, and ρ = 0.35, *p* = 0.001 for days 28 and 56, respectively). Early immune reconstitution above median values of NK cells was significantly associated with improved relapse-free survival (HR 2.84 [95% CI 1.29–6.28], *p* = 0.01, and HR 4.19 [95% CI 1.68–10.4], *p* = 0.002, for day 28 and 56, respectively). Early concentrations above the median value of the mature effector CD56dim NK cell subset were significantly associated with decreased relapse incidences at 1 year, 7% (95% CI 1.8–17) vs. 28% (95% CI 15–42), *p* = 0.04, and 7% (95% CI 1.8–18) vs. 26% (95% CI 14–40) %, *p* = 0.03, for days 28 and 56, respectively. The results suggest a protective effect of high doses of NK cells in grafts and during early immune reconstitution and support the perception of NK cells as innate effector cells with anti-tumor effects in the setting of allogeneic stem cell transplantation.

## Introduction

Although allogeneic hematopoietic stem cell transplantation (HSCT) is a potentially curative treatment for various hematologic malignancies, it is still a procedure associated with a substantial risk of disease relapse and transplant-related morbidity and mortality (TRM) including graft-vs.-host disease (GVHD) (1–3). The stem cell graft not only contains stem cells that establish donor derived hematopoiesis, but also mature immune cells responsible for early elimination of residual tumor cells through the graft-vs.-tumor (GVT) effect (4, 5). Early lymphocyte immune reconstitution is associated with improved clinical outcomes after transplantation (6). The contents of the stem cell grafts impacts immune reconstitution (7), yet few studies report data from both stem cell grafts and immune reconstitution. T lymphocytes from the adaptive immune system are essential actors in the allo-recognition involved in both GVT effects and GVHD (8–10). However, graft contents and immune recovery of T cells do not always reflect outcomes after transplantation (11–14). NK cells are the first lymphocytes that return to normal concentrations post-transplant (15), and separate studies report improved clinical outcomes of both high graft doses (16, 17) and robust immune reconstitution (14, 18) of NK cells. NK cells recognize self from non-self through a series of inhibitory and activating receptors engaged by the major histocompatibility complex (MHC) class 1 specific killer Ig-like receptors (KIRs) (19). Moreover, NK cells are capable of lysing and eliminating tumor cells of both leukemias and solid tumors through non-major histocompatibility complex (MHC)-specific activating receptors of the natural killer (NK) and natural cytotoxicity receptor (NCR) families (20, 21). MICA and MICB (MHC class 1 chain-related proteins A and B) and ULBPs (UL16-binding proteins) expressed by “stressed” cells (e.g., from infection, autoimmunity, malignant transformation) are able to engage the activating receptor NKG2D and trigger NK cell-mediated tumor elimination through perforin-meditated cytotoxicity, interleukin-priming (IL-12,15,18), and stimulation of costimulatory receptors (22, 23). Though they are considered potent innate effector cells in the setting of HSCT (24–26), little is known about the NK cell graft composition and its possible impact on immune reconstitution after transplantation.

Here we report data on stem cell grafts, immune reconstitution, and clinical outcomes in HSCT patients transplanted with PBSC. Details on immune reconstitution and the impact on clinical outcomes for the included patients is recently published as part of a larger cohort (27) why results in this report are focused on stem cell grafts and their impact on immune reconstitution and clinical outcomes.

## Materials and Methods

Our original study included 111 patients transplanted with peripheral blood stem cells or bone marrow and data on immune reconstitution and clinical outcomes in all patients are recently published (27). Here, data on healthy donors together with data from stem cell grafts in patients transplanted with PBSC are presented and correlated with data on immune reconstitution and clinical outcomes. New results include characterization of cells in stem cell grafts which were analyzed for correlation to immune reconstitution as well as clinical outcomes. Analyzes of stem cell donors were included for comparisons of immune reconstitution after transplantation to healthy controls. A subset of the included stem cell donors were donors for included recipients and these pairs were investigated for associations between peripheral blood cell concentration in donors and cell doses in their subsequent harvested stem cell grafts.

The study was approved by the Danish National Committee on Health Research Ethics (H-15005137), and all participants gave written informed consent prior to transplantation in accordance with the Declaration of Helsinki.

### Patients

Ninety patients transplanted with PBSC at the Bone Marrow Transplantation Unit, Department of Hematology, Copenhagen University Hospital, Rigshospitalet were consecutively included in the period between October 2015 and March 2017. Only patients above 18 years receiving their first transplantation were included. Two patients had incomplete graft sample data and were excluded from analyses. Characteristics for the included 88 patients are shown in Table 1.

**Table 1:** Patient and transplant characteristics.

**N**	**88**
Follow-up time, days, median, min-max	667 (386–884)
Age, years, median, min-max	60 (23–74)
**Disease**, ***n*****, percent**	
AML ALL MDS Myelofibrosis NHL Chronic leukemia Other	34 (39%) 9 (10%) 25 (28%) 8 (9%) 5 (6%) 3 (3%) 4 (5%)
**Disease risk index**	
Low Intermediate High	7 (8%) 72 (82%) 9 (10%)
**Donor**, ***n*****, percent**	
MRD MUD	23 (26%) 65 (74%)
**Donor HLA match**, ***n*****, percent**	
10/10 or 9/10 allele match 1 Ag mismatch	81 (92%) 7 (8%)
**Donor-recipient sex**, ***n*****, percent**	
M/M M/F F/F F/M	43 (49%) 20 (23%) 16 (18%) 9 (10%)
**CMV serological status**	
–/– –/+ +/+ +/–	22 (25%) 8 (9%) 33 (38%) 25 (28%)
**Conditioning intensity**, ***n*****, percent**	
Myeloablative Non-myeloablative	36 (41%) 52 (59%)
**Conditioning regimen**, ***n*****, percent**	
TBI-Flu Flu-Treo TBI-Cy TBI-Etopophos Other	50 (57%) 25 (29%) 10 (11%) 2 (2%) 1 (1%)

*AML, acute myeloid leukemia; MDS, myelodysplastic syndrome; ALL, acute lymphoblastic leukemia; BM, bone marrow; PBSC, peripheral blood stem cells; MRD, matched related donor; MUD, matched unrelated donor; TBI, total body irradiation; Flu, fludarabine; Treo, treosulfan; Cy, cyclophosphamide*.

#### Conditioning Regimen

Myeloablative regimens were cyclophosphamide 120 mg/kg plus 12 Gy total body irradiation (TBI); cyclophosphamide was replaced with Etophophos 18,00 mg/m2 for acute lymphoblastic leukemia. Cyclophosphamide doses were calculated using adjusted ideal body weight if BMI was >27.5. Patients ≤ 65 years with myelodysplastic syndrome (MDS) were conditioned with Fludarabine 150 mg/kg plus Treosulfan 42 g/m2. Ten patients transplanted with PBSC and myeloablative conditioning received anti-thymocyte-globulin (ATG) Grafalon 10 mg/kg at day−3,−2, and−1. Non-myeloablative conditioning was Fludarabine 90 mg/m^2^ plus 2 Gy TBI; TBI was increased to 4 Gy in patients not previously treated with chemotherapy.

#### GVHD Prophylaxis and Diagnosis

Patients treated with myeloablative conditioning received cyclosporine 6.25 mg/kg or tacrolimus 0.06 mg/kg orally b.i.d. from day−1 combined with a short-course of intravenous methotrexate on days 1 (15 mg/m^2^), 3, 6, and 11 (10 mg/m^2^). Cyclosporine or tacrolimus was tapered to stop at day 180, unless GVHD was present. Non-myeloablatively treated patients received tacrolimus 0.06 mg/kg orally b.i.d. from day−3 combined with mycofenolate mofetil 15 mg/kg b.i.d. from day 0 to days 27 in related transplants. In HLA matched unrelated transplants, tacrolimus 0.06 mg/kg orally b.i.d from day−3 was combined with mycophenolate mofetil 15 mg/kg orally t.i.d. from 0 to 30, then b.i.d. until day 40 and tapered to stop day 96 in the absence of GVHD. Tacrolimus was tapered from day 56 to 0 by day 180 in related non-myeloablative transplants, and from day 100 to zero by day 180 in unrelated non-myeloablative transplants in the absence of GVHD. In antigen mismatched non-myeloablative transplants, cyclosporine 5 mg/kg orally b.i.d., and sirolimus 2 mg orally q.d. from day−3 and mycofenolate mofetil 15 mg/kg t.i.d. from day 1 was administered. Cyclosporine was tapered to stop day 180 and mycofenolate mofetil day 150; sirolimus was tapered to stop day 365, unless GVHD was present.

Acute and chronic GVHD (aGVHD, cGVHD) were diagnosed and graded from clinical symptoms and biopsies according to the modified Glucksberg-Seattle criteria (28, 29).

#### Relapse

Relapse was morphologically diagnosed in leukemia patients as more than 5% blast cells in the bone marrow or the appearance of extramedullary leukemic lesions, in MDS patients, recurrence of MDS by morphology, cytogenetics, or both. Relapse in lymphoma patients was defined by new or progressing foci on PET/CT scans.

### Healthy Donors and Stem Cell Donors

To compare concentrations and composition of cell subsets in stem cell grafts and during patient immune reconstitution, 53 healthy donors were included for parallel analyses. These were all matched related stem cell donors and peripheral blood samples were collected during the screening process about 30 days prior to mobilization with G-CSF. Seventeen of these 50 donors were donors for the included patients and were assessed for correlations between donor peripheral blood lymphocyte concentrations and graft concentrations. Donors were mobilized with filgrastim (Nivestim Hospira UK Limited, Maidenhead, UK) 10 μg/kg/day for 5 days prior to leukapheresis; one donor received additional plerixafor (Mozobil Sanofi-Aventis, Denmark) 0.24 mg/kg between day 1 and 2 of leukapheresis.

### Sample Collection After Transplantation

Graft samples of 500 μL were obtained from the donor graft bag and analyzed within 8 h. For analyses of immune reconstitution patient samples were analyzed at day 28 (median 28 [min, max 23–39], 56 (median 56 [min, max 48–76]), 91 (median 91 [min, max 74–108]), 180 (median 181 [min, max 166–239]), and 365 (median 365 [min, max334–452]) after transplantation. Patient samples were collected on 2 mL EDTA tubes and analyzed within 24 h.

### Absolute Cell Concentrations and Immune Phenotyping

Samples from donors, stem cell grafts and patients during immune reconstitution were analyzed using the same method. Absolute concentrations of total CD3, CD4, and CD8 T cells and CD16/CD56 (antibody-mix same fluorochrome) NK cells were evaluated by flow cytometry with BD™ Trucount tubes containing fluorescent beads as an internal standard according to the manufacturer's instructions (BD Biosciences, San Jose, California). Residual volume was used for immune phenotyping in a 2-tube, stain-lyse,10-color flow cytometry panel developed for the study. For multi-fluorochrome staining, 100 μL of whole blood was labeled for TCRαβ-FITC, TCRγδ-PE, CD4-PerCP-Cy5.5, CD45RA-PE-Cy7, CD197-APC, CD45RO-APC-H7, HLA-DR-V450, CD3-V500, and CD8-BV605 for tube 1, and TCRVδ2-FITC, TCRγδ-PE, TCRVδ1-PE-Cy7, CD314-APC, CD16-APC-H7, CD56-V450, CD3-V500, and CD337-BV605 for tube 2. Cell subset fractions and concentrations were calculated from the absolute concentrations of total CD3-, CD4-, and CD8- T cells and CD16/56 NK cells obtained from the TruCount analyses.

#### Acquisition and Data Analysis

Samples were analyzed using BD™ FACSCanto flow cytometer with the BD™ FACSDiva software which was also used for data analyses. Phenotype subset definition and gating strategies are shown in Supplemental Figure 1, Supplemental Table 1.

#### Analyses of Cell Subsets in Donors, Stem Cell Grafts, and During Immune Reconstitution

Absolute transplanted doses and concentrations of CD3-, CD4-, CD8-, and TCR γδ T cells, total NK cells and two NK cell subsets were analyzed for associations between graft contents, immune reconstitution, and clinical outcomes. Based on CD16 and CD56 expression NK cells at different stages of development were defined (30): immature CD16lowCD56bright (termed CD56bright) cells and mature effector CD16posCD56dim (termed CD56dim) cells. Doses of CD34 cells, white blood cell counts, and lymphocytes were additionally analyzed in grafts. For immune characterization, we analyzed fractions of differentiation subsets in terms of naïve (CD45RA+CD197+), central memory (CD45RA-CD197+), effector memory (CD45RA–CD197–) and terminally differentiated effector memory (TEMRA, CD45RA+CD197–) cells of CD4-, CD8-, and TCR γδ T cells. The fractions of the CD56dim and CD56bright subsets within total NK cells together with the fraction of TCR γδ cells within total CD3 T cells and fractions of the Vδ1, Vδ2, and nonVδ1, nonVδ2 within total TCR γδ cells, were also analyzed. Expression of the activating receptor NKG2D on the two defined NK cell subsets was reported as the mean fluorescence intensity (MFI) and analyzed for associations to the primary outcome.

In grafts, cells transplanted cell doses were 10^6^/kg. Throughout the text, “concentrations” refers to absolute concentrations (10^6^/L) and “percentages” or “fractions” refer to percent of the specified cell subsets of the specified cell populations. Cell doses and concentrations were analyzed as categorical variables (high vs. low) dichotomized by the median value for all analyses.

### Outcome Definitions

Graft cell contents were analyzed for associations to immune reconstitution and clinical outcomes. Associations between early immune reconstitution and clinical outcomes are reported in brief. Time was calculated from day 0, 28, and 56, respectively, for investigating associations to the stem cell graft, day 28, and 56 immune reconstitution. The primary outcome was relapse-free survival (RFS) which was defined as the probability of survival without relapse with an event defined as the composite of death and/or relapse. Secondary outcomes included relapse, aGVHD, and cGVHD. The same time points were used for analyses of relapse incidence and cGVHD. For associations between immune reconstitution and aGVHD, the earliest sample after transplantation (day 28) was used. Nine patients were diagnosed with aGVHD before their respective day 28 sample and were therefore excluded from the aGVHD analyses. For all outcomes, patients with graft rejection (*n* = 1) and graft failure (*n* = 2) were censored at the time of rejection or booster transplantation. Correlations between peripheral blood cell concentrations of stem cell donors and graft doses, and grafts doses and early immune reconstitution were analyzed. Graft contents and immune reconstitution of T and NK cells day 28–365 were characterized and compared to cell subset concentrations and distribution in healthy donors. The expression of the activating receptor NKG2D (CD314) was included for analyses on the defined NK cell subsets.

### Statistical Analyses

Kaplan Meier survival analyses and Cox proportional hazards models were used to investigate the associations between graft doses/concentrations during early immune reconstitution and RFS. In addition to cell doses/concentrations, pre-transplant factors thought to have a possible impact on RFS were included in the analyses. Disease Risk Index was included according to previously published criteria (31). Pre-transplant factors significant in univariate analyses were included in the multivariable models. Cumulative incidence of relapse, aGVHD, and cGVHD were compared using Gray's test for competing risks (32) with death from other causes than the studied event as competing event. Only grade II-IV aGVHD were included in the aGVHD analyses. Due to the patient number multivariate analyses were performed only for RFS. Spearman correlation was used for non-parametric testing of associations between graft cell doses and early immune reconstitution and for associations between concentrations of peripheral blood of the stem cell donors and graft cell doses. The Wilcoxon signed rank test was used for paired testing of associations between the cell fractions in grafts and by the time of early immune reconstitution. The paired sample *T*-test was used to assess changes in the expression of NKG2D on NK cells throughout the first year after transplantation. The independent *T*-test was used to compare differences in the expression of NKG2D on NK cells between groups. Statistical analyses were performed using SPSS version 22 (SPSS, Chicago, IL) and R version 3.2.0 (R Foundation for Statistical Computing, Vienna, Austria) combined with the EZR platform (33). *P* ≤ 0.05 were considered statistically significant.

## Results

### Patient Outcome

After a median of 667 (386–884) days 66 of 88 (75%) patients were alive. Eight patients (9%) died from relapse and 14 patients (16%) died from TRM. In the TRM group, 4 patients died from aGVHD, three patients died from organ failure, three patients died from infection, two patients died from toxicity, one patient died from cGVHD, and one patient died from unknown causes other than relapse later than 2 years after transplantation. Two and four patients died from TRM prior to their day 28/56 samples. Relapse-free survival in all patients was 57% (95% CI 39–71%), TRM was 13% (95% CI 7–22%) and relapse incidence was 26% (95% CI 12–43%). A total of 17 (19%) patients relapsed during the observation time. Twenty-eight patients (32%) experienced grade II-IV aGVHD, and 46 (52%) experienced cGVHD.

### Graft Characteristics and Immune Reconstitution

**Figure 1:**
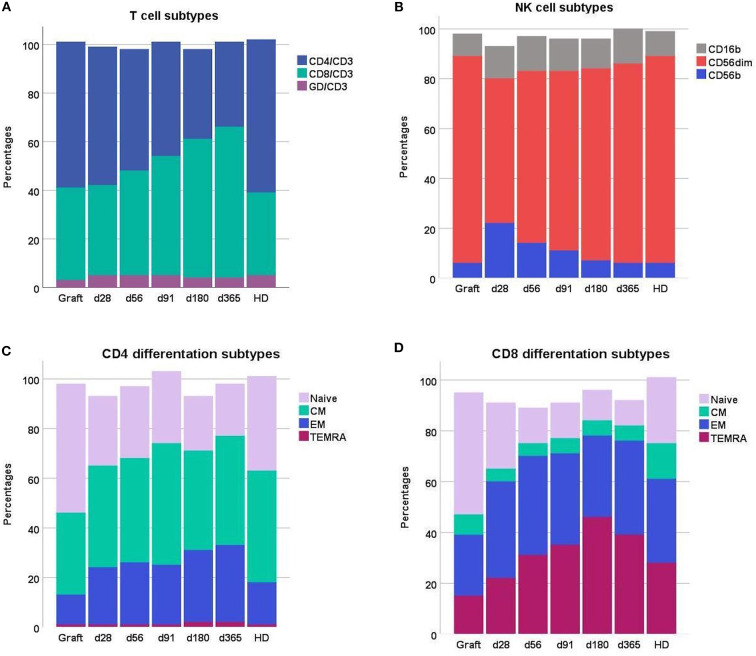
Distribution of **(A)** T cell subsets and **(B)** NK cell subsets **(C)** CD4 differentiation subsets and **(D)** CD8 differentiation subsets in grafts, peripheral blood of patients day 28–365 after transplantation and peripheral blood of healthy donors (HD). Patient numbers day 28 *n* = 86, day 56 *n*=84, day 91 *n* = 79, day 180 *n* = 71, day 365 *n* = 53; grafts *n* = 88, HD *n* = 53. Each compartment is constructed by the use of median percentages. CM, central memory; EM, effector memory; TEMRA, terminally differentiated effector memory.

The absolute and relative doses of T and NK cells in the PBSC grafts, in patients during day 28–356 immune reconstitution and in healthy donors are presented in Table 2, Figure 1. The median dose of transplanted cells was 8 (6.3–10) × 10^6^ cells/kg for CD34 cells, 887 (731–1162) × 10^6^ cells/kg for total white blood cells and 317 (240–408) × 10^6^ cells/kg for total lymphocytes. Fractions of naïve cells in both the CD4- and CD8 T cell compartment were higher in G-CSF stimulated PBSC grafts compared both to patients during immune reconstitution and healthy controls. The total NK cell concentration was higher in patients day 28 post-transplant compared to healthy donors (*p* < 0.001 for comparison of means) and though decreasing 1–3 months post-transplant, still exceeded that of healthy donors 1 year post-transplant (*p* < 0.001 for comparison of means). The fractions of the CD56bright subset represented more than 20% of total NK cells 28 days post-transplant and decreased to values comparable to healthy donors (median 6.3%, IQR 4.5–9.1%) 1-years post-transplant, Figure 1B.

**Table 2:** T and NK cell concentrations and subsets in grafts, peripheral blood in patients 28–365 days after transplantation and peripheral blood in healthy donors (HD).

**Cell subset concentration, median (IQR)**	**Graft, *N* = 88 10^**6**^/kg**	**Day 28, *N* = 86 10^**6**^/L**	**Day 56, *N* = 84 10^**6**^/L**	**Day 91, *N* = 79 10^**6**^/L**	**Day 180, *N* = 71 10^**6**^/L**	**Day 365, *N* = 53 10^**6**^/L**	**HD, *N* = 53 10^**6**^/L**
T cells (CD3)	255 (193–330)	420 (248–575)	425 (260–738)	460 (280–880)	880 (550–1,600)	1,200 (750–2,000)	1,500 (1,150–1,700)
CD4	157 (109–192)	205 (150–333)	200 (140–328)	230 (120–320)	330 (220–450)	440 (310–515)	930 (715–1,100)
CD8	94 (66–126)	160 (71–240)	175 (100–395)	220 (99–490)	470 (210–1,000)	690 (400–1,500)	345 (430–630)
TCR γδ	7.1 (4.5–14)	22 (7–57)	22 (8.8–59)	24 (8.4–61)	41 (13–99)	70 (22–117)	41 (25–81)
NK cells (CD16/56)	27 (20–36)	285 (150–403)	200 (143–348)	160 (100–300)	250 (140–410)	270 (160–380)	200 (140–300)
CD56dim	23 (16–30)	162 (86–262)	128 (85–215)	112 (71–192)	159 (100–293)	198 (122–302)	165 (113–250)
CD56bright	1.7 (1.1–2.6)	60 (32–98)	34 (17–64)	22 (13–34)	17 (12–33)	16 (8.8–27)	14 (8.9–20)
**Relative concentrations, %**							
CD4/CD3	60 (53–66)	57 (46–63)	50 (32–62)	47 (29–57)	37 (23–52)	35 (24–47)	65 (54–71)
CD8/CD3	38 (33–42)	37 (31–50)	43 (34–59)	49 (34–63)	57 (46–71)	62 (48–75)	32 (27–42)
CD4/CD8, ratio	1.6 (1.2–2.0)	1.6 (0.9–2.0)	1.1 (0.6–1.9)	1.0 (0.4–1.6)	0.6 (0.3–1.1)	0.6 (0.3–1.1)	2.0 (1.3–2.7)
CD56dim/NK	83 (77–88)	59 (48–71)	69 (57–78)	72 (59–83)	77 (66–84)	80 (71–86)	83 (78–87)
CD56bright/NK	6.2 (4.3–8.5)	22 (15–32)	14 (10–23)	11 (8.2–19)	7.3 (4.6–13)	5.7 (2.9–11)	6.3 (4.5–9.1)
Naïve CD4/CD4	52 (44–61)	28 (20–39)	29 (17–38)	29 (18–37)	22 (14–33)	21 (14–33)	38 (28–49)
Central memory CD4/CD4	33 (28–40)	41 (35–49)	42 (34–49)	40 (34–49)	40 (32–48)	44 (34–48)	43 (36–53)
Effector memory CD4/CD4	12 (8.6–17)	23 (17–31)	25 (16–35)	24 (18–35)	29 (22–42)	31 (21–40)	18 (10–20)
TEMRA CD4/CD4	0.5 (0.3–1.2)	0.8 (0.4–1.8)	1.2 (0.6–2.4)	1.3 (0.6–2.4)	1.6 (1.0–5.1)	1.7 (0.9–4.7)	0.5 (0.2–1.8)
Naïve CD8/CD8	48 (35–61)	26 (9.7–37)	14 (4.9–33)	14 (7.3–27)	12 (6.8–22)	10 (5.3–18)	24 (12–37)
Central memory CD8/CD8	8.1 (5.4–11)	5.3 (3.0–8.8)	4.8 (2.4–9.2)	5.6 (3.6–9.5)	5.7 (3.1–9.8)	5.6 (2.2–9.2)	13 (9.8–18)
Effector memory CD8/CD8	24 (16–31)	38 (26–51)	39 (30–52)	36 (27–47)	32 (24–41)	37 (28–52)	31 (23–40)
TEMRA CD8/CD8	15 (10–26)	22 (13–40)	31 (18–44)	35 (23–51)	46 (33–57)	39 (24–56)	29 (12–42)
Naïve TCR γδ/TCR γδ	7.4 (4.6–13)	1.8 (0.9–4.0)	1.8 (0.8–3.7)	2.2 (0.8–4.1)	2.1 (1.0–4.8)	2.1 (0.7–5.2)	3.6 (2.1–8.4)
Central memory TCR γδ/TCR γδ	11 (6.9–16)	1.1 (0.4–2.2)	0.9 (0.4–2.7)	0.9 (0.3–2.2)	1.2 (0.3–3.7)	2.0 (0.7–3.7)	7.8 (3.9–13)
Effector memory TCR γδ/TCR γδ	42 (31–54)	48 (33–67)	47 (30–62)	38 (23–54)	35 (22–50)	44 (28–64)	34 (23–60)
TEMRA TCR γδ/TCR γδ	35 (23–50)	46 (29–62)	49 (35–66)	52 (40–68)	60 (40–73)	47 (27–62)	43 (25–67)
TCR γδ/ CD3 T	2.7 (1.8–5.4)	5.0 (3.0–12)	4.9 (2.5–11)	4.5 (2.0–10)	3.8 (2.3–7.5)	3.9 (2.1–7.8)	2.7 (2.0–5.8)
Vδ1/ TCR γδ	24 (11–34)	10 (3.6–25)	11 (3.8–21)	12 (3.2–27)	21 (9.0–42)	31 (11–51)	26 (12–42)
Vδ2/ TCR γδ	68 (50–83)	87 (69–95)	86 (68–95)	84 (64–96)	71 (41–90)	61 (34–85)	65 (39–83)
NonVδ1–non-Vδ2/ TCR γδ	6.1 (2.5–9.9)	2.0 (1.0–5.8)	2.1 (0.7–5.1)	2.7 (0.9–42)	3.5 (0.9–9.0)	3.9 (1.2–12)	6.0 (2.5–16)

Table 3 show correlations between transplanted cells/kg and peripheral blood cell concentrations early after transplantation in patients. Except from the CD56bright NK subset, all transplanted cell doses correlated significantly with concentrations in patients 28 after transplantation. However, paired analyses of cell subset fractions in stem cell grafts and during early immune reconstitution (Table 3, Supplemental data) showed significant differences in all cell subsets except CD8 cells out of total T cells by day 28 and effector memory TCRγδ cells out of total TCRγδ cells by day 56. We also tested if the transplanted CD34 cell dose correlated to concentrations of the major cell subsets during early immune reconstitution. The transplanted CD34 cell dose was not significantly correlated to patient day 28 or 56 T cell concentrations (Spearman correlation *p* = 0.26 and *p* = 0.99), CD4 T cell concentrations (*p* = 0.09 and *p* = 0.49), CD8 T cell concentrations (*p* = 0.66, and *p* = 0.88) or NK cell concentrations (*p* = 0.62 and *p* = 0.98).

**Table 3:** Correlations between transplanted cell T and NK cell doses (10^6^/kg) and peripheral blood concentrations (10^6^/L) in patients 28 and 56 days after transplantation, Spearman's ρ and *p*-values.

**Cell subset**	**Day 28**, ***n*** **=** **86**		**Day 56**, ***n*** **=** **84**	
	**ρ**	***p*-value**	**ρ**	***p*-value**
CD3 CD4 CD8 TCR γδ	0.38 0.42 0.32 0.57	< 0.001 < 0.001 0.003 < 0.001	0.17 0.30 0.10 0.28	0.12 0.006 0.37 0.01
NK cells (CD16/56) CD56dim CD56bright	0.26 0.35 0.0	0.02 0.001 0.55	0.35 0.36 0.08	0.001 0.001 0.48

### NKG2D Expression on NK Cells

**Figure 2:**
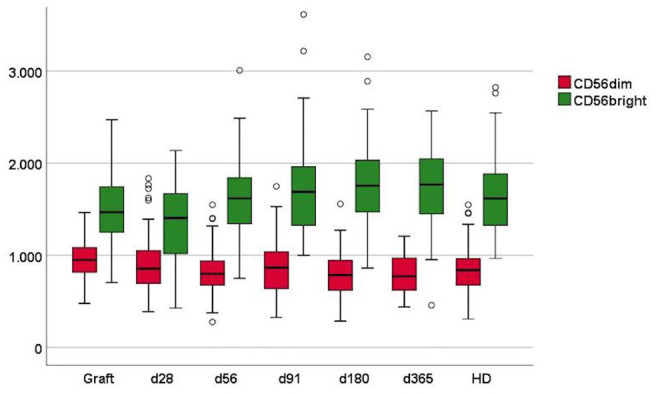
Mean fluorescence intensity (MFI) of NKG2D in NK cell subsets in grafts, peripheral blood of patients day 28–365 after transplantation and in peripheral blood of healthy donors (HD). Patient numbers day 28 *n* = 86, day 56 *n* = 84, day 91 *n* = 79, day 180 *n* = 71, day 365 *n* = 53, grafts *n* = 88, HD *n* = 53. Whiskers represent interquartile range.

The expression of NKG2D was higher in the CD56bright subset compared to the CD56dim subset in grafts and healthy donors as well as during immune reconstitution in patients, Figure 2. When comparing groups, the NKG2D expression on CD56bright cells was significantly lower in patients day 28 post-transplant compared to healthy donors (median MFI 1404 vs. 1644, *p* < 0,00) but generally increased and was not significantly different from healthy donors 1-year post-transplant (median MFI 1767 vs. 1644, *p* = 0.25). The NKG2D expression in patients generally increased in the CD56bright subset and decreased in the CD56dim subset from day 28 through 365 after transplantation (Supplemental Table 4). In paired analyses, the increase of NKG2D on the CD56bright subset was significant from day 28 to 56, and the decrease on the CD56dim subset was significant from day 91 to 180 post-transplant (Supplemental Table 5). The NKG2D expression in stem cell grafts were significantly higher compared to healthy donors in the CD56dim subset (median MFI 951 vs. 854, *p* = 0.01), while it was significantly lower on the CD56bright subset (median MFI 1467 vs. 1644, *p* = 0,05). The expression of NKG2D on CD56dim and CD56bright NK cell subtypes in the graft and during immune reconstitution was analyzed in relation to RFS (Supplemental Table 6). The only significant association was in patients with high NKG2D expression on CD56dim cells day 56 who had an increased risk of death or relapse compared to patients with low NKG2D expression.

### Stem Cell Donor Peripheral Blood Cell Concentrations and Correlation to Graft Concentrations

In the 17 available donor/graft pairs, we tested if peripheral concentrations of major cell subsets in stem cell donors before G-CSF stimulation were correlated to graft concentrations. There were significant correlations for total T cells, CD8 T cells, and NK cells (Spearman correlation *p* = 0.01, *p* = 0.001 and *p* = 0.03, respectively), and a similar trend in CD4 T cells, *p* = 0.07. Supplemental Figure 2 displays the correlation for NK cells.

### Relapse-Free Survival

Recipient and donor age, disease type, donor type, HLA-match, conditioning regimen, the use of ATG, recipient, and donor CMV status and disease risk index were analyzed for univariate correlation to RFS (**Table 7**, Supplemental data). HLA mismatch and donor age above 30 years were significantly associated with an increased risk of death or relapse. These were included in multivariate analyses of the impact of cell doses in the graft and in peripheral blood concentrations during immune reconstitution on RFS. Disease risk index failed to predict RFS, perhaps due to low numbers of patients in the low and high risk groups.

#### Transplanted Cell Doses

**Figure 3:**
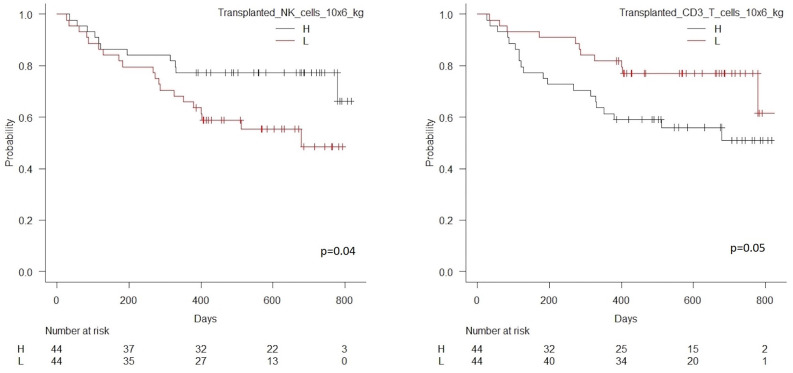
Kaplan Meier estimated relapse-free survival in patients transplanted with high vs. low transplanted NK cell doses (median 27 × 10^6^/kg), *n* = 88, *p* = 0.04, left hand side, and high vs. low transplanted CD3 T cell doses (median 255 × 10^6^/kg), *n* = 88, *p* = 0.05, right hand side.

**Figure 4:**
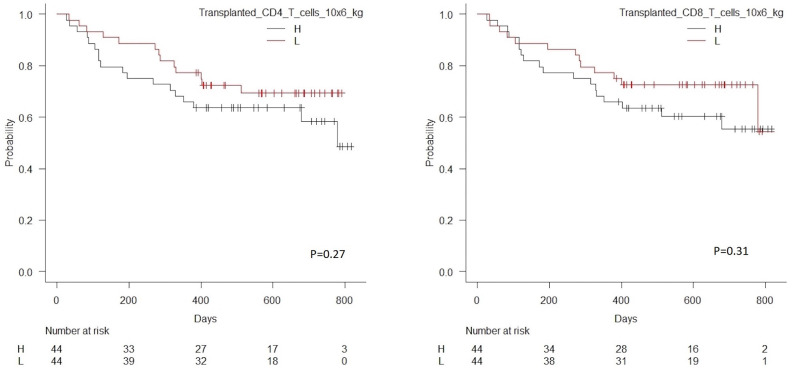
Kaplan Meier estimated relapse-free survival in patients transplanted with high vs. low transplanted CD4 T cell doses (median 157 × 10^6^/kg), *n* = 88, *p* = 0.27, left hand side, and high vs. low transplanted CD8 T cell doses (median 94 × 10^6^/kg), *n* = 88, *p* = 0.31, right hand side.

Patients transplanted with an NK cell dose below the median of 27 × 10^6^/kg had significantly higher risk of death or relapse compared to patients transplanted with an NK cell dose above this value, HR 2.12 (95% CI 1.01–4.45), *p* = 0.04, Table 4A. In contrast, patients transplanted with a CD3 T cell dose below the concentration of 255 × 10^6/^kg had significantly lower risk of death or relapse compared to patients transplanted with a CD3 T cell dose above this value, HR 0.47 (95%CI 0.23–0.99), *p* = 0.05. Figure 3 shows estimated survival by dose of transplanted CD3 T and NK cells. The transplanted dose of NK and CD3 T cells remained significantly associated with RFS in multivariate analyses including HLA match and donor age, Table 4B. Due to the association between high transplanted doses of CD3 T cells and decreased RFS, we included ATG in the multivariate analyses of the impact of CD3 T cell doses; a low CD3 dose remained significantly associated with decreased risk of death or relapse, HR 0.40 (95% CI 0.19–0.84), *p* = 0.02, when HLA match, donor age and the use of ATG were included in multivariate analysis. Doses of transplanted CD4 T cell and CD8 T cells were not significantly associated to RFS, Figure 4.

**Table 4:** **(A)** Univariate and **(B)** multivariable analyses of the impact of transplanted 10^6^ cells/kg on relapse-free survival, *n* = 88.

**Transplanted cell subset, 10^**6**^/kg**	**HR (95% CI)**	***p*-value**
**A)**		
CD34		
High	1.00	
Low	0.75 (0.37–1.53)	0.42
WBC		
High	1.00	
Low	0.81 (0.40–1.65)	0.57
Lymphocytes		
High	1.00	
Low	0.93 (0.46–1.88)	0.84
CD3 T		
High	1.00	
Low	0.47 (0.23–0.99)	0.05
CD4 T		
High	1.00	
Low	0.67 (0.33–1.36)	0.27
CD8 T		
High	1.00	
Low	0.69 (0.34–1.41)	0.31
TCR γδ		
High	1.00	
Low	1.42 (0.70–2.87)	0.34
NK (CD16/56)		
High	1.00	
Low	2.12 (1.01–4.45)	0.04
CD56dim		
High	1.00	
Low	1.24 (0.61–2.52)	0.56
CD56bright		
High	1.00	
Low	0.88 (0.43–1.79)	0.72
**Variable**	**HR (95% CI)**	***p*****-value**
**B)**		
Transplanted NK cells (below vs. above 27 × 10^6^kg)	2.50 (1.17–5.37)	0.02
Donor age (below vs. above 30 years)	2.24 (1.06–4.73)	0.03
HLA match (9–10/10 vs. other)	3.12 (1.15–8.47)	0.03
Transplanted CD3 T cells (below vs. above 255 × 10^6^kg)	0.41 (0.19–0.87)	0.02
Donor age (below vs. above 30 years)	2.38 (1.12–5.05)	0.02
HLA match (9–10/10 vs. other)	2.68 (1.01–7.10)	0.05

*Transplanted cell subsets are dichotomized by median values. Hazard ratio is for death or relapse. WBC, white blood cell count*.

#### Early Immune Reconstitution

**Figure 5:**
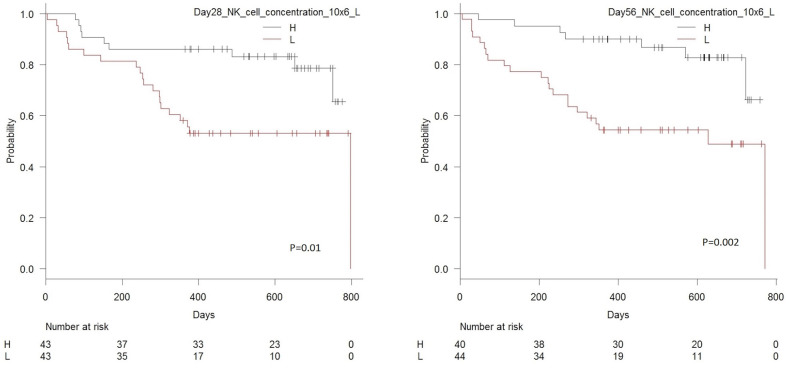
Kaplan Meier estimated relapse-free survival in patients with high vs. low concentrations (median 285 × 10^6^/L) of NK cells in peripheral blood 28 days after transplantation, *n* = 86, *p* = 0.01 (left-hand side), and high vs. low concentrations (median 200 × 10^6^/L) of NK cells in peripheral blood 56 days after transplantation, *n* = 84, *p* = 0.002 (right-hand side).

Tables 5A,B shows univariate and multivariate associations between cell subset concentrations in peripheral blood during immune reconstitution day 28 and 56 after transplantation and RFS for NK cells. As shown, low day 28 concentrations of total NK cells, the CD56dim subset and the CD56bright subset were associated with significantly higher risk of death or relapse. By day 56, low concentrations of total NK cells and the CD56dim subset remained significantly associated with increased risk of death or relapse. Association between day 28 and 56 NK cell concentrations ad RFS are shown in Figure 5. Associations for T cell subsets were similar to previously published data (27).

**Table 5:** **(A)** univariate analyses of the impact of NK cell subset concentrations in peripheral blood 28 and 56 days after transplantation on relapse-free survival and **(B)** multivariate analyses of significant NK cell subsets adjusted for significant pre-transplant factors (HLA-match and donor age).

**Cell subset concentration**	**Day 28**		**Day 56**	
	**HR (95% CI)**	***p*-value**	**HR (95% CI)**	***p*-value**
**A)**				
NK cells (CD16/56)				
High	1.00		1.00	
Low	2.84 (1.29–6.28)	0.01	4.19 (1.68–10.4)	0.002
CD56dim				
High	1.00		1.00	
Low	2.84 (1.28–6.28)	0.01	3.56 (1.50–8.43)	0.004
CD56bright				
High	1.00		1.00	
Low	4.35 (1.85–10.2)	0.001	1.61 (0.75–3.46)	0.23
**B)**				
NK cells (CD16/56)				
High	1.00		1.00	
Low	2.81 (1.26–6.25)	0.01	4.25 (1.70–10.7)	0.002
CD56dim				
High	1.00		1.00	
Low	3.03 (1.35–6.80)	0.007	3.30 (1.36–8.00)	0.008
CD56bright				
High	1.00			
Low	3.92 (1.64–9.35)	0.002		

*Cell concentrations are dichotomized at the median value. Hazard ratio is for death or relapse*.

### Relapse Incidence

#### Transplanted Cell Doses

**Figure 6:**
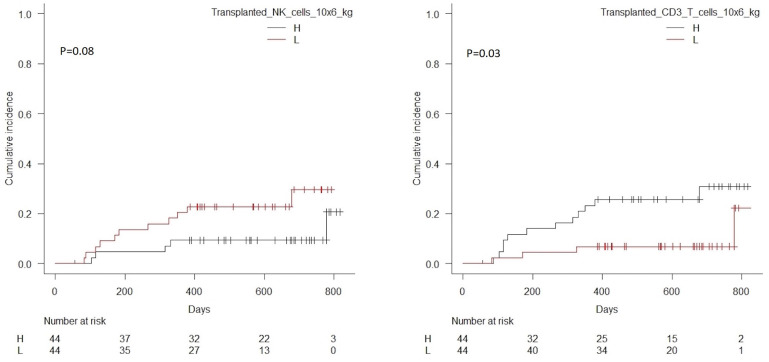
Cumulative incidence of relapse with death from transplant-related-mortality as a competing event in patients transplanted with high vs. low doses of transplanted NK cells (median 27 × 10^6^/kg), *p* = 0.08, left hand side, and CD3 T cells (median 255 × 10^6^/kg), *p* = 0.03, right hand side.

**Figure 7:**
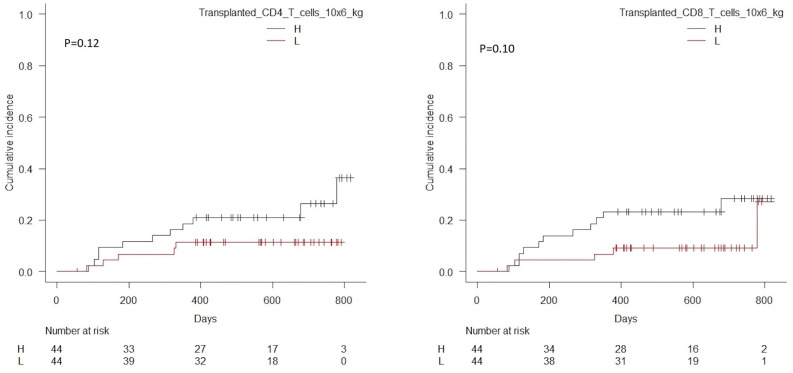
Cumulative incidence of relapse with death from transplant-related-mortality as a competing event in patients transplanted with high vs. low doses of transplanted CD4 T cells (median 157 × 10^6^/kg), *p* = 0.12, left hand side, and CD8 T cells (median 94 × 10^6^/kg), *p* = 0.10, right hand side.

Patients with transplanted CD3 T cell doses above the median value of 255 × 10^6^kg had significantly higher cumulative incidence of relapse compared to patients transplanted with doses below this value, *p* = 0.03, Table 6. Patients transplanted with NK cell doses below the median value of 27 × 10^6^kg had a trend toward a higher cumulative incidence of relapse compared to patients transplanted with doses above this value, *p* = 0.08. Correlations for NK and CD3 cells are shown in Figure 6. No significant associations were seen for CD4 or CD8 T cells, Figure 7.

**Table 6:** Cumulative incidence of relapse with death from transplant-related mortality as a competing event in patients transplanted with high vs. low cell doses of analyzed cell subsets, p-values from Gray's test for competing risks.

**Transplanted cell subset, 10^**6**^/kg**	***p*-value**
CD34 WBC Lymphocytes	0.31 0.07 0.26
CD3 T CD4 T CD8 T TCR γδ	0.03 0.12 0.10 0.65
NK (CD16/56) CD56dim CD56bright	0.08 0.22 0.42

#### Early Immune Reconstitution

**Figure 8:**
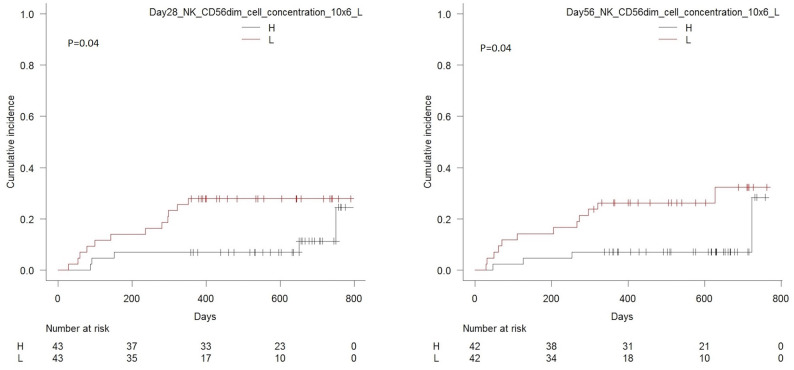
Cumulative incidence of relapse with death from transplant-related-mortality as a competing event in patients with high vs. low concentrations of CD56dim NK cells (median 162 × 10^6^/L) in peripheral blood 28 days after transplantation, *n* = 86, *p* = 0.04 (left-hand side) and patients with high vs. low concentrations of CD56dim NK cells (median 128 × 10^6^/L) in peripheral blood 56 days after transplantation, *n* = 84, *p* = 0.04 (right-hand side).

Robust day 28 immune reconstitution of the NK CD56dim subset was significantly associated with decreased risk of relapse incidence with one year relapse incidence of 7% (95% CI 1.8–17%) in patients with concentrations above the median value of 162 × 10^6^ cells/L compared to 28% (95% CI 15–42%) in patients with concentrations below this value, *p* = 0.04. By day 56, patients with concentrations above the median value of total NK cells (*p* = 0.04) and CD56dim cells (*p* = 0.03) had lower cumulative incidence of relapse compared to patients with values below the median of these cell types, Figure 8. Associations to remaining cell subsets where similar to those presented for the original cohort (27).

### GVHD

#### Transplanted Cell Doses

No dose of any of the analyzed cell subtypes were significantly associated with the incidence of acute or chronic GVHD, Table 7.

**Table 7:** Cumulative incidence of **(A)** acute and **(B)** chronic GVHD with death from other causes as a competing event in patients transplanted with high vs. low cell doses of analyzed cell subsets, *p*-values from Gray's test for competing risks.

**Transplanted cell subset, 10^**6**^/kg**	***p*-value**
**A)**	
CD34 WBC Lymphocytes	0.94 0.45 0.55
CD3 T CD4 T CD8 T TCR γδ	0.59 0.61 0.78 0.48
NK (CD16/56) CD56dim CD56bright	0.75 0.73 0.08
**B)**	
CD34 WBC Lymphocytes	0.73 0.75 0.97
CD3 T CD4 T CD8 T TCR γδ	0.44 0.86 0.87 0.27
NK (CD16/56) CD56dim CD56bright	0.13 0.32 0.12

#### Early Immune Reconstitution

There was a trend toward an increased incidence of aGVHD in patients with day 28 CD56bright NK cell concentration below the median of 60 × 10^6^/L, *p* = 0.08. Associations to remaining cell subsets where similar to those presented for the original cohort (27).

## Discussion

The overall strength of this study was the simultaneous characterization of T and NK cells in both donors, grafts and during long term immune reconstitution. Our results show significant associations between high doses of graft NK cells and robust early NK cell immune reconstitution and increased RFS after HSCT in patients transplanted with PBSC. During early immune reconstitution, patients with high concentrations of the CD56dim NK subset had significantly decreased relapse incidence compared to patients with low concentrations. NK cells were the only analyzed cell subset significantly associated to improved clinical outcomes regarding graft doses as well as during early immune reconstitution and the associations remained significant in multivariate analyses.

Graft-derived NK cells are primary effectors of early graft-vs.-tumor effect in haplo-identical *ex vivo* CD3 or TCRαβ cell depleted grafts, and recent studies confirms the efficiency of this transplant variant (34–36). There are only a few studies on the possible effect of graft NK cell doses on the risk of relapse in HLA-matched transplants and with varying results (12, 37–40). Results from a recent study by Maggs et al. (16) elegantly demonstrated decreased relapse rates in 107 patients transplanted with PBSC with high NK cell concentrations primarily correlated to the CD56dim subset expressing high levels of the activating receptor DNAM. Though the study by Maggs et al. was conducted in a T-cell depleted setting and the majority of the patients in our study (89%) received a T-cell replete transplantation, the results indicate similar protection from disease relapse by NK donor cells in an HLA-matched transplant setting. Conflicting results from studies investigating the clinical impact of immune reconstitution of NK cells at different time points and in different transplant settings have been reported (13, 14, 41, 42). Previous studies did report an impact on relapse (41, 42), while a more recent study from our group (14) showed associations between day 28 high concentrations of total NK cells and OS but with no effect on relapse. This could be due to missing NK cell subtyping in our 2016 study, as the day 28 concentration of total NK cells in the current study was also not significantly associated with relapse incidence and only the CD56dim subset showed significant association to this outcome. The total NK cell concentrations 28 and 56 days post-transplant were significantly associated to RFS as was the day 28 concentrations of the CD56bright subset. As reported, the CD56bright subset represents a more immature phenotype with regulatory properties and high proliferation rates while the CD56dim subsets signifies a mature phenotype with cytolytic activity (24, 30). This could be in accordance with our findings that this effector subset could be more active in the graft-vs.-tumor effect leading to lower relapse incidence in patients with high concentrations post-transplant, while there was a trend toward lower incidence of aGVHD in patients with high concentration of the “regulatory” CD56bright subset. Further receptor profiling and functional assays would be necessary to support this hypothesis and the possible role of NK cells in GVHD remains controversial (43).

Overall, there were significant associations between transplanted doses of the main cell types (CD3, CD4, CD8, NK) and the corresponding cell concentrations in patients during early immune reconstitution. However, paired analyses of subset fractions within the main cell types showed significant differences between fractions in grafts and during immune reconstitution suggesting an altered and graft independent distribution already early after transplantation. For NK cells, there was a significant correlation between transplanted graft NK cell doses and early NK cell immune reconstitution, while there was no correlation to the transplanted dose of CD34 cells. This could suggest that the expansion of mature NK cells within the graft contributed to the peripheral regeneration. However, doses of transplanted CD56brigth NK cells did not correlate with concentrations of CD56bright NK cells during early immune reconstitution and there were no significant associations in paired analyses between the percentual distribution of the CD56bright subset in the graft and at day 28 and 56 after transplantation. Therefore, the early post-transplant increased fraction of the immature CD56bright subset, independent of the fraction in the graft, could suggest that development from a graft progenitor cell might have contributed to the overall increased number of total NK cells at this time point. Interestingly, a recent study found NK cell reconstitution in day 21 post-transplant bone marrow samples to be associated with overall survival and non-relapse mortality, indicating an important role of what is probable progenitor-derived NK cells in or from the bone marrow microenvironment (44). Further characterization in search of the somewhat elusive NK cell progenitor is necessary to elaborate on these findings (45, 46).

The expression of the activating receptor NKG2D on NK cell subsets in the HSCT setting is only reported in few previous studies (16, 47–49). As Mags et al. (16) we could not demonstrate associations between the NKG2D expression in graft NK cells and relapse nor did we find association during immune reconstitution. In our study the highest expression of NKG2D was found on the CD56bright NK subset in grafts as well as in peripheral blood of healthy donors and patients during post-transplant immune reconstitution. This was somewhat unexpected as NKG2D is normally associated with cytotoxicity responses primarily exhibited by a more mature NK cell phenotype (47). As others (48, 49) we found that the NKG2D expression on the CD56bright subset generally increased during the first year after transplantation, however in contrast, the expression in transplant patients early after HSCT was significantly lower compared to healthy donors in this study. This could be explained by NKG2D receptor down-regulation upon constant stressing stimuli in the context of inflammation, danger-signals, and up-regulation of MIC ligands during the transplant course (47, 50). Increased levels of soluble NKG2D ligands and cytokines known to reduce NKG2D levels (TGF-β) could also be contributing factors (51). The significant differences in the NKG2D expression in the different NK cell subtypes in grafts compared to healthy donors is to our knowledge, not earlier reported and could be caused by the influence of G-CSF which, however, was not analyzed in this study.

We found a somewhat unexpected association between transplanted doses above the median of 255 × 10^6^/kg CD3 T cells and reduced RFS and increased relapse incidence; most studies report unchanged or improved outcomes in patients transplanted with high CD3 cell doses (12, 52, 53). However, this study was comparable to recent practice regarding donor stimulation regimen and doses of transplanted T cells (12), and results remained significant after adjustment for ATG in the conditioning regimen which could be expected to alter the risk due to *in vivo* T cell depletion post-transplant. Regarding T cell immune reconstitution, the association between TCR γδ cells and improved RFS found in patients in this study are similar to results from our original study of immune reconstitution in 108 transplant patients (27) and will not be further elaborated here.

The concurrent findings of improved RFS in patients with both high transplanted doses and early high immune reconstitution of NK cells substantiates the proposed ability of NK cells to participate in mediating the graft-vs.-tumor effect leading to protection from relapse after transplantation (24, 54). We here included descriptive analyses of the expression of the NKG2D receptor on NK cells during the course of HSCT while functional analyses and effector cytokine profiling of NK cells were not a part of the study. Further analyses of activating and inhibitory receptor of the NK and NCR family together with KIR typing may provide further insights to innate and allogeneic NK cell mechanism involved in possible cytolytic effect on residual malignant cells after HSCT (20, 21, 55). In addition, the relative heterogenous patient population in terms of disease type and conditioning regimen was a weakness to this study.

The correlation between NK cell concentrations in stem cell donors before stimulation and graft doses and subsequently between graft doses and post-transplant immune reconstitution reported here opens for the possibility of identifying donors who are likely to mobilize grafts containing high NK cell doses with the potential of reducing relapse risk as many patients have more than one suitable donor (56). The relationship between cell concentrations in screening of peripheral blood in donors and cell doses in graft products should be analyzed in a larger study population to confirm our findings and, if so, to identify a relevant cut-off for optimal graft contents.

In conclusion, results from this study showed improved RFS with a lower risk of disease relapse in patients transplanted with high graft doses and early robust immune reconstitution of NK cells. Since donor NK concentrations before G-CSF mobilization correlated to graft concentrations which again correlated to immune reconstitution, immune profiling of stem cell donors, and graft contents could be beneficial in pre-transplant risk assessment as both donor cells and graft contents are modifiable factors in HSCT (57). Results from this study support ongoing research in the development of new methods for activating and modifying NK cells for relapse prevention in order to improve survival in transplant patients (58).

## Data Availability Statement

The datasets generated for this study are available on request to the corresponding author.

## Ethics Statement

The studies involving human participants were reviewed and approved by Danish National Committee on Health Research Ethics (H-15005137). The patients/participants provided their written informed consent to participate in this study.

## Author Contributions

LM performed the research, analyzed data, and wrote the paper. HM and LR contributed with new reagent and analytic tools in the laboratory. NA, IS, LF, and EH performed research. BK and SP performed research and helped write the paper and analyze data. HM, AF-N, and HS designed the research. HS and HM furthermore performed research and analyzed data.

## Conflict of Interest

The authors declare that the research was conducted in the absence of any commercial or financial relationships that could be construed as a potential conflict of interest.
