# Supplementary material for: Improved Relapse-Free Survival in Patients With High Natural Killer Cell Doses in Grafts and During Early Immune Reconstitution After Allogeneic Stem Cell Transplantation
Source: Front Immunol. 2020 May 29;11:1068. doi: 10.3389/fimmu.2020.01068 (PMC7273963; doi:10.3389/fimmu.2020.01068)
Supplement: Supplementary file 1 [file Data_Sheet_1.PDF]

Supplemental data

Table 1. Two-tube panel of monoclonal antibodies (mAB) used for leukocyte phenotyping. Antibodies were titrated and used in saturating concentrations.

Flurochrome	mAB	Clone	Manufacturer catalog no.
**Tube 1**			
FITC	TCRαβ	Clone WT31	BD-333140
PE	TCRγδ	Clone 11F2	BD-333141
PerCp Cy5.5	CD4	Clone SK3	BD-332772
PE-Cy7	CD45RA	Clone L48	BD-337186
Alexa Fluor 647	CD197	Clone 150503	BD-560816
-			
APC-H7	CD45RO	Clone UCHL1	BD-561137
V450	HLA-DR	Clone L243	BD-655874
V500	CD3	Clone SP34-2	BD-560770
BV605	CD8	Clone SK1	BD-564116
**Tube 2**			
FITC	TCRVδ2	Clone 123R3	Miltenyi-130-095-798
PE	TCRγδ	Clone 11F2	BD-333141
-			
PE-Vio770	TCRVδ1	Clone REA173	Miltenyi-130-100-540
APC	CD314	Clone 1D11	BD-558071
-			
APC-H7	CD16	Clone 3G8	BD-560195
V450	CD56	Clone B159	BD-560360
V500	CD3	Clone SP34-2	BD-560770
BV605	CD337	Clone p30-15	BD-563384

Figure 1. Extracts from flow panels and gating strategies in tube 1 (page 3-4) and tube 2 (page 5-6). Dead cells/debris and dublets were remowed as shown in the dot plot forward and side scatter (FSC/SSC) and FSC Area/FSC High. Lymphocytes were identified based on their forward and side scatter properties. In tube 1, CD3pos events were selected on the CD3/SSC dot plot and subsequintly CD3 T cells were separated in a TCR αβ/TCR γδ plot and TCR αβ T cells were further separated in a CD4/CD8 plot (not shown). TCR αβ T cells, TCR γδ T cells and CD4- and CD8 T cells were separately investigated for differentation markers in a CD45RA/CD45RO plot for identification of CD45RAneg/CD45RApos memory cell phenotypes, and a CD45RA/CD197 plot for identification of central(CD45RAnegCD197pos)/effector(CD45RAneg/CD197neg) memory-, CD45RApos/CD197pos naive-, and CD45RApos/CD197neg TEMRA cell phenotypes. The expression of HLA-DR were investigated in histograms for TCR αβ T cells, TCR γδ T cells and CD4- and CD8 T cells separately. In tube 2, TCR γδ (CD3pos) T cells were identified in a CD3/TCR γδ plot, and TCR γδ T cells were furthermore separated in subtypes in a TCR Vδ2/TCR Vδ1 plot. NK cells were identified in a CD56/CD16 plot gated on CD3neg lymphocytes and two populations (CD56dim and CD56bright) were identified based on CD16- and CD56 expression. TCR γδ T cells, TCR Vδ1 T cells, TCR Vδ2 T cells, CD56dim NK cells and CD56bright NK cells were separately investigated for the expression of CD314 and CD337 in histograms and analyzed based on fractions of cells positive as well as the MFI (mean fluorescence intensity) expression.

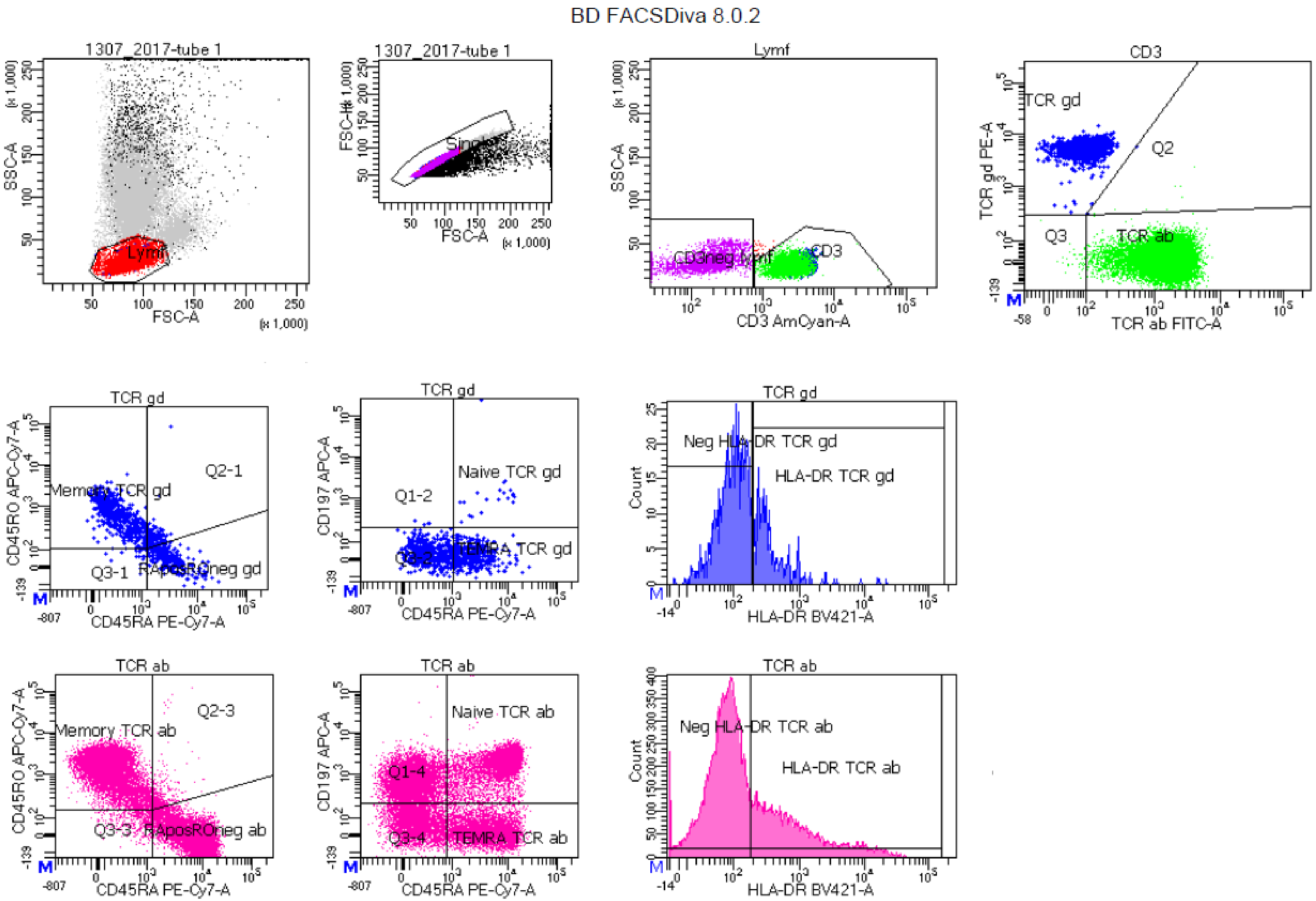
BD FACSDiva 8.0.2
1307_2017-tube 1
SSC-A
FSC-A
Lymf
FSC-H
Singlets
Lymf
CD3neg Lymf
CD3
CD3 AmCyan-A
TCR gd
Q2
Q3
TCR ab
TCR gd PE-A
TCR ab FITC-A
Memory TCR gd
Q2-1
Q3-1
RAposROneg gd
CD45RO APC-Cy7-A
CD45RA PE-Cy7-A
Q1-2
Naive TCR gd
TEMRA TCR gd
CD197 APC-A
Neg HLA-DR TCR gd
HLA-DR TCR gd
Count
HLA-DR BV421-A
TCR ab
Memory TCR ab
Q2-3
Q3-3
RAposROneg ab
Q1-4
Naive TCR ab
Q3-4
TEMRA TCR ab
Neg HLA-DR TCR ab
HLA-DR TCR ab

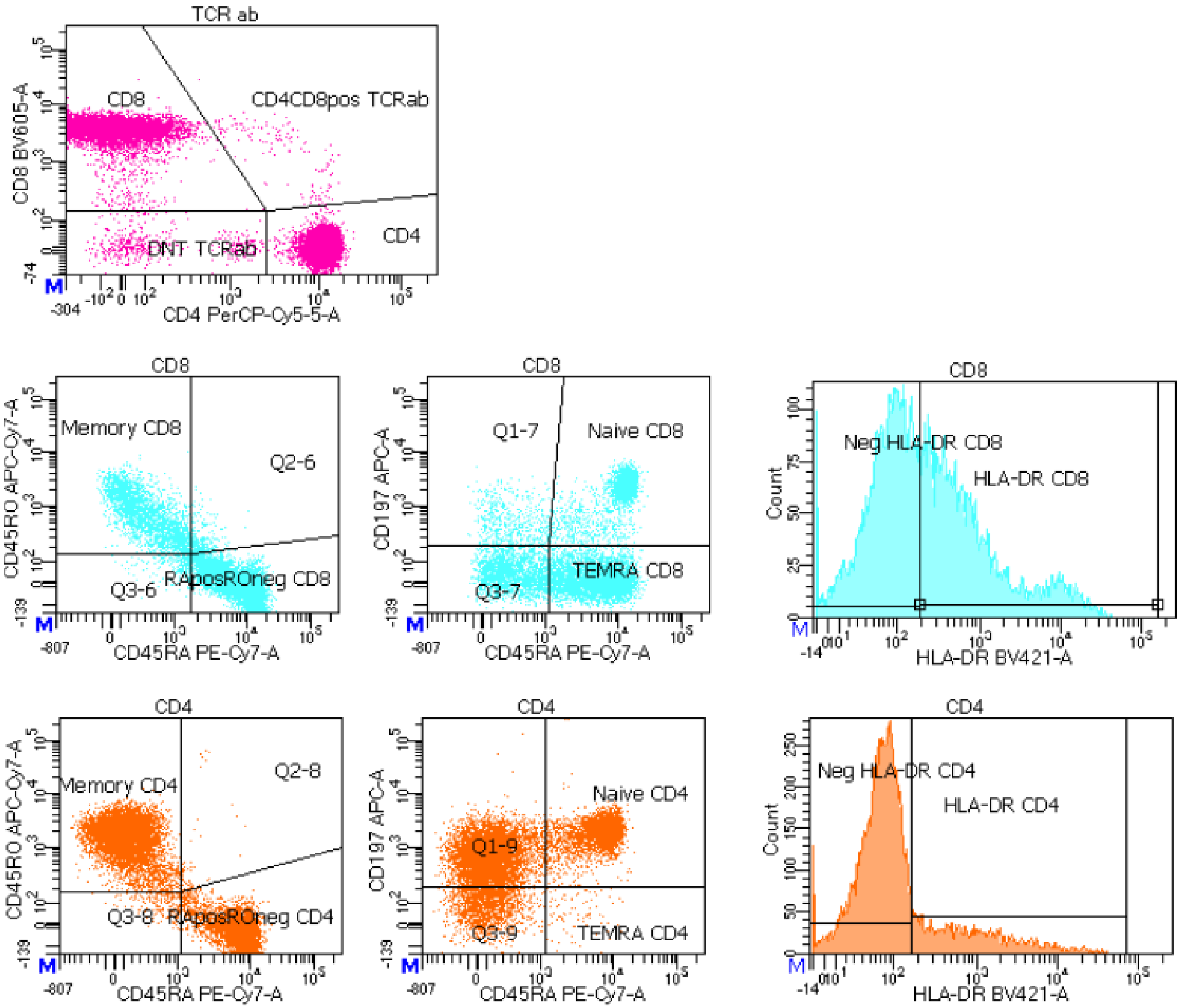
TCR ab
CD8
CD4CD8pos TCRab
DNT TCRab
CD4
CD8 BV605-A
CD4 PerCP-Cy5-5-A
CD8
Memory CD8
Q2-6
Q3-6
RAposROneg CD8
CD45RO APC-Cy7-A
CD45RA PE-Cy7-A
CD8
Q1-7
Naive CD8
Q3-7
TEMRA CD8
CD197 APC-A
CD45RA PE-Cy7-A
CD8
Neg HLA-DR CD8
HLA-DR CD8
Count
HLA-DR BV421-A
CD4
Memory CD4
Q2-8
Q3-8
RAposROneg CD4
CD45RO APC-Cy7-A
CD45RA PE-Cy7-A
CD4
Naive CD4
Q1-9
Q3-9
TEMRA CD4
CD197 APC-A
CD45RA PE-Cy7-A
CD4
Neg HLA-DR CD4
HLA-DR CD4
Count
HLA-DR BV421-A

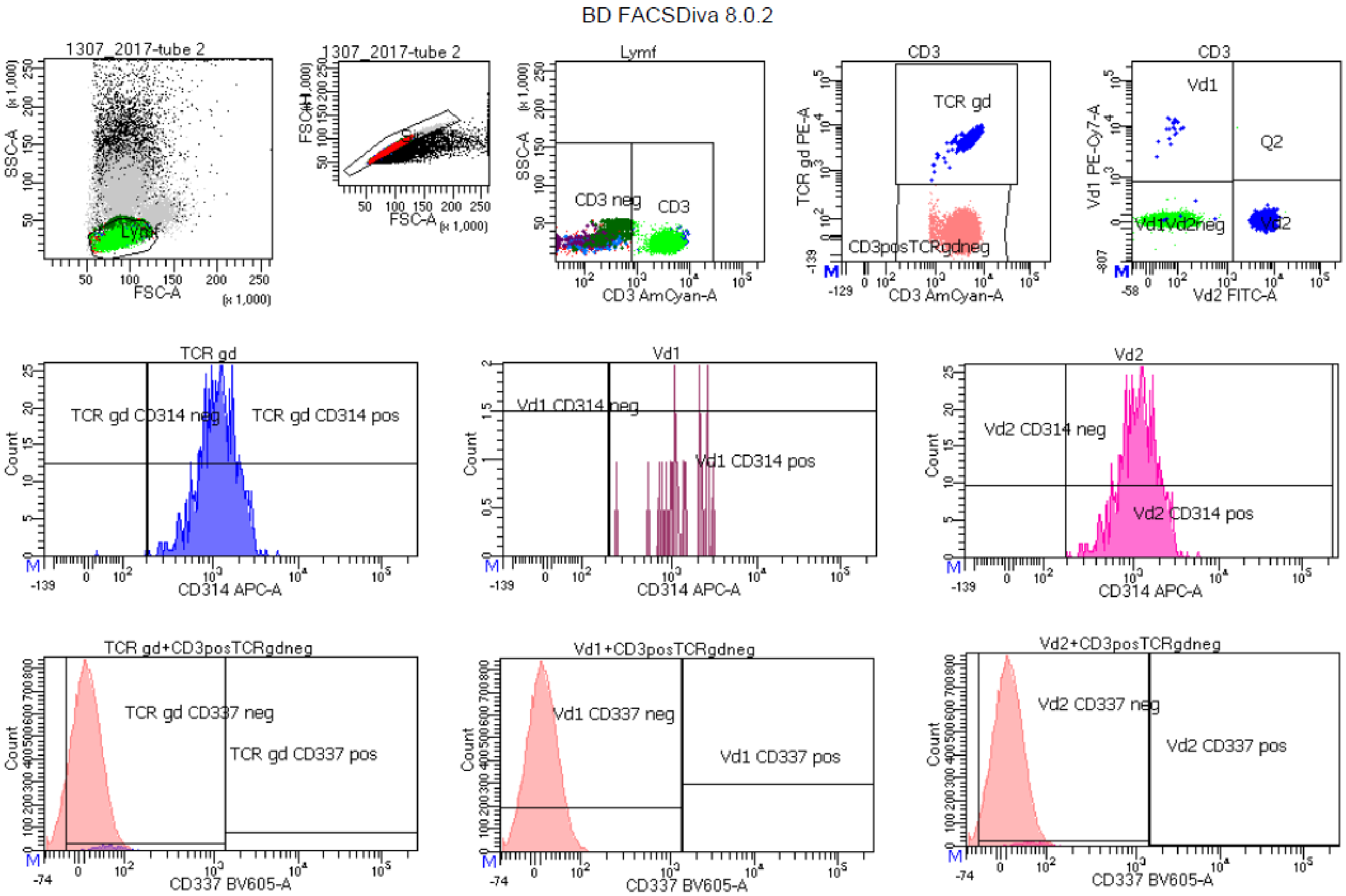
BD FACSDiva 8.0.2
1307_2017-tube 2
SSC-A
FSC-A
Lymf
1307_2017-tube 2
FSC-H
FSC-A
Lymf
CD3 neg
CD3
SSC-A
CD3 AmCyan-A
CD3
TCR gd
CD3posTCRgdneg
TCR gd PE-A
CD3 AmCyan-A
CD3
Vd1
Q2
Vd1Vd2neg
Vd2
Vd1 PE-Cy7-A
Vd2 FITC-A
TCR gd
TCR gd CD314 neg
TCR gd CD314 pos
Count
CD314 APC-A
Vd1
Vd1 CD314 neg
Vd1 CD314 pos
Count
CD314 APC-A
Vd2
Vd2 CD314 neg
Vd2 CD314 pos
Count
CD314 APC-A
TCR gd+CD3posTCRgdneg
TCR gd CD337 neg
TCR gd CD337 pos
Count
CD337 BV605-A
Vd1+CD3posTCRgdneg
Vd1 CD337 neg
Vd1 CD337 pos
Count
CD337 BV605-A
Vd2+CD3posTCRgdneg
Vd2 CD337 neg
Vd2 CD337 pos
Count
CD337 BV605-A

BD FACSDiva 8.0.2

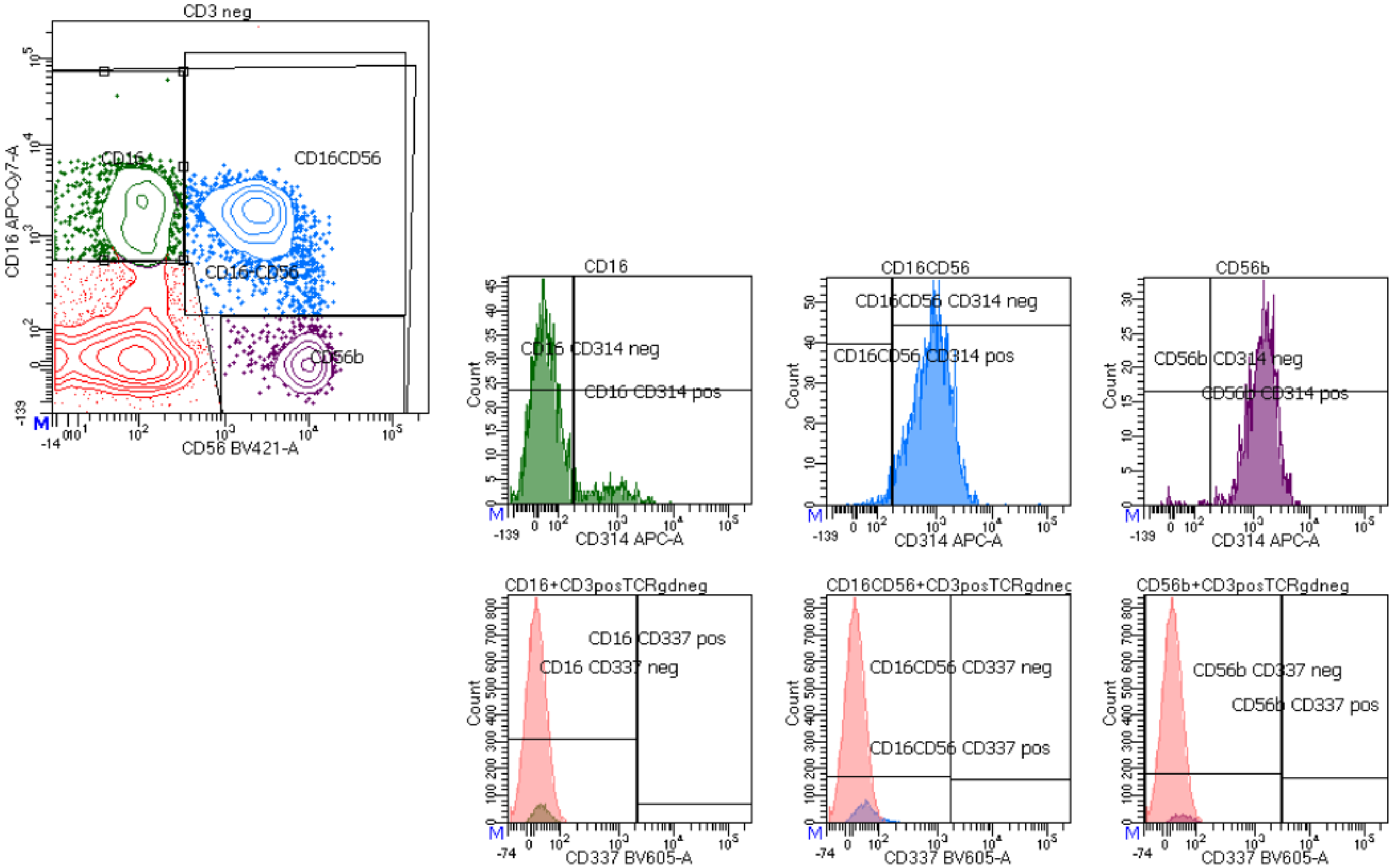

Table 2. Subset definitions and phenotypes. The absolute concentrations of CD3, CD4, CD8 and NK cells were calculated by the BD ™ Trucount system. Additional fractions and concentrations were calculated from the panels in Table 1.

Subset definition	Phenotype
CD3+TCR γδ+	TCR γδ cells
CD3+TCR γδ+CD45RA+CD197+	Naive TCR γδ cells
CD3+TCR γδ+CD45RA-CD197+	Central memory TCR γδ cells
CD3+TCR γδ+CD45RA-CD197-	Effector memory TCR γδ cells
CD3+TCR γδ+CD45RA+CD197-	TEMRA TCR γδ cells
CD3+TCR γδ+Vδ1+	TCR Vδ1 cells
CD3+TCR γδ Vδ2+	TCR Vδ2 cells
CD3+TCR γδ+Vδ1-Vδ2-	TCR nonVδ1-nonVδ2
CD3+TCR αβ+CD4+	CD4 T cells
CD3+TCR αβ+ CD4+CD45RA+CD197+	Naive CD4 T cells
CD3+TCR αβ+ CD4+CD45RA-CD197+	Central memory CD4 T cells
CD3+TCR αβ+ CD4+CD45RA-CD197-	Effector memory CD4 T cells
CD3+TCR αβ+ CD4+CD45RA+CD197-	TEMRA CD4 T cells
CD3+TCR αβ+CD8+	CD8 T cells
CD3+TCR αβ+ CD8+CD45RA+CD197+	Naive CD8 T cells
CD3+TCR αβ+ CD8+CD45RA-CD197+	Central memory CD8 T cells
CD3+TCR αβ+ CD8+CD45RA-CD197-	Effector memory CD8 T cells
CD3+TCR αβ+ CD8+CD45RA+CD197-	TEMRA CD8 T cells
CD16/CD56+	NK cells
CD16+CD56++	CD56bright NK cells
CD16++CD56+	CD56dim NK cells

Table 3. Paired analyses of cell subset fraction in stem cell grafts and in patients day 28 and 56 after transplantation, p-values from Wilcoxon's signed rank test.

Cell subset	Day 28, n=86	Day 56, n=84
CD4/CD3	*0.02*	*<0.001*
CD8/CD3	0.29	*<0.001*
CD56dim/NK	*<0.001*	*<0.001*
CD56bright/NK	*<0.001*	*<0.001*
TCR γδ/ CD3	*<0.001*	*<0.001*
Vδ1/ TCR γδ	*<0.001*	*<0.001*
Vδ2/ TCR γδ	*<0.001*	*<0.001*
Naive CD4/CD4	*<0.001*	*<0.001*
Central memory CD4/CD4	*<0.001*	*<0.001*
Effector memory CD4/CD4	*<0.001*	*<0.001*
TEMRA CD4/CD4	*<0.001*	*<0.001*
Naive CD8/CD8	*<0.001*	*<0.001*
Central memory CD8/CD8	*<0.001*	*<0.001*
Effector memory CD8/CD8	*<0.001*	*<0.001*
TEMRA CD8/CD8	*<0.001*	*<0.001*
Naive TCR γδ/TCR γδ	*<0.001*	*<0.001*
Central memory TCR γδ/TCR γδ	*<0.001*	*<0.001*
Effector memory TCR γδ/TCR γδ	*0.004*	0.45
TEMRA TCR γδ/TCR γδ	*<0.001*	*<0.001*

Table 4. Median Fluorescent Intensity (MFI) of NKG2D expression on CD56dim and CD56bright NK cell subsets in stem cell grafts and during day 28-365 immune reconstitution, median (IQR).

NK cell subset	Graft, N=88	Day 28, N=86	Day 56, N=84	Day 91, N=79	Day 180, N=71	Day 365 ,N=53
CD56dim	951 (809-1087)	854 (691-1051)	798 (675-941)	865(634-1045)	786 (614-951)	771 (620-971)
CD56bright	1467 (1241-1749)	1404 (1010-1674)	1619 (1327-1845)	1690 (1307-1961)	1756 (1460-2038)	1767 (1448-2067)

Table 5. Paired comparison of the development of MFI values on CD56dim and CD56bright NK cell subsets in patients through the first year after transplantation, p-values from paired samples T-test.

NK cell subset	Day 28-56, N=84	Day 56-91, N=79	Day 91-180, N=71	Day 180-365, N=53
CD56dim	P= 0.129	P=0.60	*P=0.03*	P=0.59
CD56bright	*P<0,001*	P=0.08	P=0.67	P=0.90

Table 6. Cox proportional model of relapse-free survival in patients dichotomized (median MFI) in groups by high versus low NKG2D expression on CD56dim and CD56bright NK cell subsets in grafts, on day 28 and day 56 after transplantation. Hazard ratio is for death or relapse.

Timepoint and NK cell subset	Risk Score/ Hazard Ratio	95% Confidence interval	P=value
Graft CD56dim, n=88			
High NKG2D	1.19	0,58-2.41	0.64
Low NKG2D	1.0		
Graft CD56bright, n=88			
High NKG2D	1.01	0.71-1.44	0.94
Low NKG2D	1.0		
Day 28 CD56dim, n=86			
High NKG2D	1.12	0.78-1.62	0.55
Low NKG2D	1.0		
Day 28 CD56bright, n=86			
High NKG2D	0.95	0.66-1.37	0.79
Low NKG2D	1.0		
Day 56 CD56dim, n=84			
High NKG2D	2,06	1,31-3,242	*0,002*
Low NKG2D	1.0		
Day 56 CD56bright, n=84			
High NKG2D	1,35	0,91-1,99	0,14
Low NKG2D	1.0		

Figure 2. Correlations of graft NK cell dose and NK cell concentrations in screening blood samples from 17 peripheral stem cell donors.

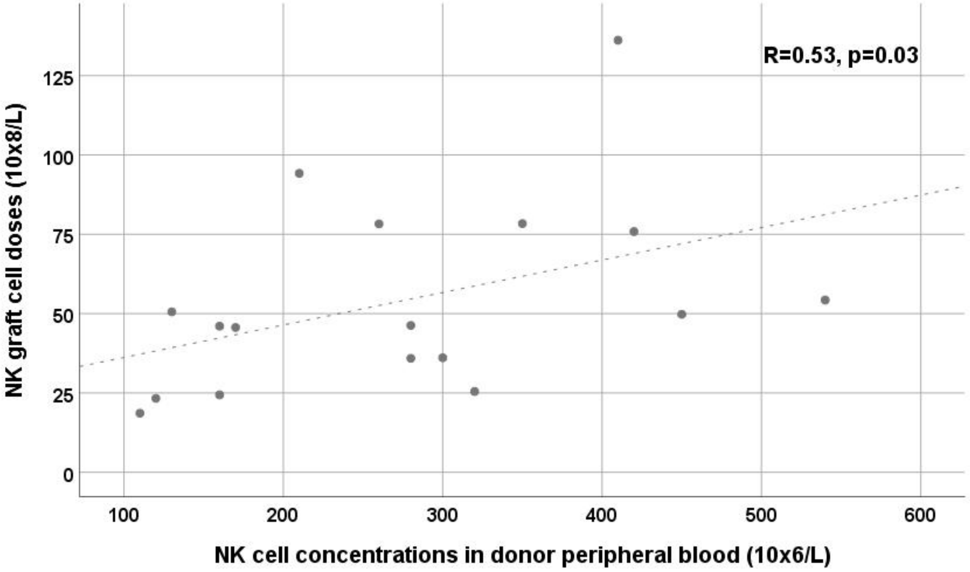

Table 7. Univariate analyses of pre-transplant factors and impact on relapse-free survival, n=88. Hazard ratio is for death or relapse. AML; acute myeloid leukemia, MDS; myelodysplastic syndrome, CMV; cytomelagovirus.

Variable	N	Relapse-free survival	
		HR (95%CI)	P-value
Recipient age			
<45	15	1.00	
>45	73	0.75 (0.26-2.14)	0.59
Disease type,			
AML/MDS	59	1.00	
Other	29	2.00 (0.98-4.07)	0.06
Donor age,			
<30 years	46	1.00	
>30 years	43	2.28 (1.09-4.77)	0.03
Donor type,			
Matched related donor	23	1.00	
Matched unrelated donor	65	1.11 (0.50-2.48)	0.80
HLA-match			
9/10 or 10/10 allele match	81	1.00	
Antigen mismatch	7	3.01 (1.15-7.90)	0.03
Conditioning regimen,			
Myeloablative	36	1.00	
Non-myeloablative	52	1.29 (0.62-2.69)	0.50
ATG,			
No	78	1.00	
Yes	10	0.70 (0.25-2.02)	0.51
CMV donor,			
Negative	47	1.00	
Positive	41	1.28 (0.63-2.60)	0.49
CMV recipient			
Negative	30	1.00	
Positive	58	0.90 (0.43-1.89)	0.78
Disease Risk Index,			
Low	7	1.00	
Intermediate	72	3.29 (0.45-20.3)	
High	9	2.46 (0.26-23.7)	0.24
